# Top-down, Bottom-up and Middle-out Strategies for Drug Cardiac Safety Assessment via Modeling and Simulations

**DOI:** 10.1007/s40495-016-0060-3

**Published:** 2016-04-05

**Authors:** Zofia Tylutki, Sebastian Polak, Barbara Wiśniowska

**Affiliations:** 1 Unit of Pharmacoepidemiology and Pharmacoeconomics, Department of Social Pharmacy, Faculty of Pharmacy, Jagiellonian University Medical College, Medyczna 9 Str., 30-688 Cracow, Poland; 2 Simcyp Ltd. (part of Certara), Blades Enterprise Centre, S2 4SU Sheffield, UK

**Keywords:** Drug safety, Proarrhythmic effect, Modelling, Simulation

## Abstract

Cardiac safety is an issue causing early terminations at various stages of drug development. Efforts are put into the elimination of false negatives as well as false positives resulting from the current testing paradigm. In silico approaches offer mathematical system and data description from the ion current, through cardiomyocytes level, up to incorporation of inter-individual variability at the population level. The article aims to review three main modelling and simulation approaches, i.e. “top-down” which refers to models built on the observed data, “bottom-up”, which stands for a mechanistic description of human physiology, and “middle-out” which combines both strategies. Modelling and simulation is a well-established tool in the assessment of drug proarrhythmic potency with an impact on research and development as well as on regulatory decisions, and it is certainly here to stay. What is more, the shift to systems biology and physiology-based models makes the cardiac effect more predictable.

## Introduction

Model-based drug development paradigm is a relatively new concept introduced to increase the pharmaceutical research and development productivity. Its main aim is to shift compound attrition from late clinical development to earlier stages, by providing more robust, data-based and clear criteria for the go/no-go decisions [1]. Therefore, reduction in cost and increase in effectiveness are expected. The effects of such activities were reported in the recent literature and have their reflection in the drug labels [2, 3]. It proves that projection presented in the 2007 PricewaterhouseCoopers report on virtual research and development (R&D) becomes reality [4]. There are multiple examples of the practical impact of the modelling and simulation approach on the new drugs fate through the clinical phase up to the regulatory decisions. The latter, namely, the regulatory level, include e.g. approval of initial human drug dosing, clinical study protocol design, pediatric drug development, DDI study waiver requests, but also a public warning for safety issues (e.g. black box label warnings), and product withdrawal from the market.

Until recently, the main reason for the black box warnings and drug withdrawals was proarrhythmic effect yet after the ICH guidelines implementation number of cases decreased [5••]. Importantly, cardiotoxicity is also a source of the problems and early terminations at various stages of the development [6]. It all suggests that proper analysis of the cardiac risk is important and challenging at the same time. Currently, the pre-clinical assessment is based on the hERG channel inhibition measurement done in the in vitro settings with the use of cell lines enriched with the heterologous genes encoding human cardiac ion channels’ proteins. Such approach helped to decrease the number of false negatives and withdraw potentially dangerous compounds, but at the same time, it increased the number of false positives (or potential false positives) which eventually narrowed already reduced pipeline with the drugs being under development due to the safety concerns. Therefore, deeper insight into the link between the drug plus human biology triggered ECG changes, and arrhythmia risk becomes crucial for all players on the market: patients, the pharmaceutical industry, and regulators [7•].

One of the elements of these efforts is to develop a Comprehensive In vitro Proarrhythmia Assay (CIPA). It aims at modifying and modernizing current nonclinical cardiac safety screening paradigm [8, 9]. Alternative screening models and methods under consideration for the CIPA initiative include stem cells utilization in which hERG (Kv11.1; IKr current) as well as other cardiac ion channels such as the fast sodium (Nav1.5; INa current) channel, persistent sodium channel (INasus), calcium (Cav1.2; ICa current) channel as well as potassium channels such as the inward rectifier (Kir2.1-2.4; IK1 current), and slow delayed rectifying (Kv7.1; IKs current) channel can be assessed in totality. The current approach is very much “hERG-centric” while the new one brings more information; therefore, a new system for its analysis and decision making becomes necessary. In silico methods offer such possibility, and the range of models is wide starting from screening methods (QSAR-based models), up to the utilization of the biophysically detailed cardiac myocyte models. The latter, namely, mathematical models of human cardiac cells, vary with regard to the level of complexity of the mathematical description of the physiology at the ion channel (Hodgkin-Huxley or Markovian notation) and cell level (single cell up to the three-dimensional heart structure). Such methods offer the possibility to incorporate variability of either stochastic or deterministic nature and further allow for the drug cardiac safety analysis at the population level and quantitative assessment of the combination of drug and nondrug-related parameters. In combination with the PBPK models used for the exposure prediction, full in vitro-in vivo extrapolation (bottom-up approach) will be possible to be implemented.

Three main modelling and simulation approaches available to be utilized include [10] (i) top-down—models built predominantly on the observed clinical data, mainly empirical with the scope of utilization narrowed down to the range of the input data, (ii) bottom-up—models based on knowledge about the human body, therefore, as mechanistic as possible, utilizing in vitro information as the input data, and (iii) middle-out—approach combining bottom-up (model) and top-down (data) and allowing for the available in vivo information utilization to determine unknown or uncertain parameters of the model. There are two necessary elements of the successful utilization of the modelling and simulation concept: information and algorithm. The first one, namely, information includes knowledge about as well as understanding of the biological processes and observations describing crucial elements of those processes. Algorithm means mathematical procedure used for the model parameters optimization. In general, it is the objective function which extrema are searched for to find the values optimal, in the sense of assumed criteria, for decision variables. The appropriate numerical algorithms allow reaching the final solution.

In the current short review, we briefly describe the area of the cardiac safety assessment and contemporary as well as potential future utilization of the modelling and simulation approach. The scope of the article is restricted to the late discovery, development as well as post approval stages. We intentionally omit most of the discovery stage; therefore, models from the cheminformatics domain which are widely used and very useful do not fit to the above-defined scope and will not be described.

## “Top-Down” PK/PD Modelling and Simulation (M and S)

Top-down modelling approaches aim to get to the system characteristics beginning with observed data. Regarding cardiac safety, ICH E-14 guidance provides recommendations to evaluate drug effect on cardiac repolarization and identify those which do not interrupt cardiac electrophysiology [11]. Following the guidelines on conducting the thorough QT/QTc study (TQT) provides ECG data that should be analyzed and interpreted properly. TQT study is a single trial carried out in healthy volunteers with the goal of identification of the drugs with a threshold pharmacologic effect on myocardial repolarization. The evaluated endpoint is QT/QTc prolongation whose value is of regulatory concern in case of exceeding 5 ms, as judged by whether the upper bound of the 95 % confidence interval around the mean effect on QTc is larger than 10 ms. Two statistical designs of TQT trials are recommended i.e. crossover (more efficient) or parallel design depending on experimental conditions [12, 13]. The treatment arms are drug in question in therapeutic and supratherapeutic doses, placebo, and active control (usually moxifloxacin).

Analysis of QT/QTc interval data recommended by ICH E14 included three steps, i.e. analyses of central tendency, categorical analyses, and drug exposure–response analysis. However, the differences between measured QT/QTc values after drug administration and at baseline may be influenced by factors other than the drug itself. Thus, statistical models come to rescue to compare time-matched mean values for significance. Amongst analyses frequently performed are an analysis of variance (ANOVA) of high applicability to different types of study design and an analysis of covariance (ANCOVA) that combines regression and ANOVA adding to the model the influence of quantitative predictor variables (covariates), uncontrolled by experimental conditions, on dependent variable [14]. Classes of models include fixed-effects, random-effects, and mixed-effects models. Bonate explained the concept of fixed, random, and mixed effects in the analysis of pharmacodynamics data [15]. Mixed models for repeated measures data from TQT studies were investigated by Schall and Ring [16]. As an example, linear mixed-effects model (LMEM) in primary analysis in a parallel group TQT study was used by Hoch et al. [17] and by Hofmann et al. [18] in statistical assessment of time-matched mean difference in change from baseline QTc between selexipag and placebo, and bitopertin and placebo, respectively. In both cases, the dependent variable was ∆QTc, random effect: subject, fixed effects: treatment, time, time-by-treatment interaction. Mixed model ANOVA applied by Morganroth [19] in crossover TQT study on betrixaban also included period, sequence, gender, and treatment-gender interaction as fixed terms. Mixed effects ANCOVA was used to analyze the time-matched change in QTc in crossover trials on retosiban [20], lenvatinib [21], umeclidinium and umeclidinium/vilanterol [22], dabigatran [23], and parallel trials on tafenoquine [24], and parallel group/crossover study on prucalopride [25]. Revised ANCOVA model besides random and fixed terms, accounted for baseline QTc as a covariate.

Apart from QT/QTc values, the data on the drug concentrations around the time of ECG assessment is gathered to establish a quantitative relationship between drug exposure and the triggered response (E-R) i.e. QTc prolongation [11]. The term “exposure” can refer to drug concentration itself as well as to any of its summary metric [26]. Traditional “top-down” PK/PD modelling and simulation that utilizes empirical or descriptive models is frequently used to conceive the concentration-response relationship (CRR). CRR is said to play a significant role in a total evidence-based assessment of the risk of QT prolongation and influence the later drug development process and the regulatory decisions [27]. Integrated PK/PD models result in the description of the effect intensity in response to a given dosing regimen in time course; thus, they may reveal the possibility of indirect drug effects on cardiac repolarization in the case of divergent results from those in central tendency analysis. At the heart of PK/PD modelling stand the assumptions of effect compartment and the relationship between the biomarker and the effector drug concentration being either linear, hyperbolic, Emax, or sigmoidal [28].

According to Stockbridge and colleagues, Emax-type models seldom describe QT/QTc data for non-arrhythmic drugs with exemplary exception to sevoflurane and sertindol [29]. However, simple linear models with assumptions of no hysteresis effect and no active metabolites frequently sufficed. Indeed, Graham et al. used a linear mixed effects model to describe the relationship between vismodegib plasma concentration and QTc prolongation after rejecting other E-R models (inter alia, Emax) as not more suitable [30]. Although not all authors tested more complicated structures to support their PD data, selecting from different linear PD models (with or without intercept) occurred to be enough. It was sufficient for mipomersen [31], lenvatinib [21], tafenoquine [24], betrixaban [19], selexipag [17], and asenapine [32]. The case of asenapine also led to a conclusion that E-R analysis can be considered more powerful analysis method alternative to an intersection-union test. Since the inter-union test may result in high false-positive rate, E-R model would serve to harmonize the results [33]. Concentration of moxifloxacin (a usual active control) in relation to induced QT prolongation was explored by both, linear model [34], and Emax model [35]. Since the calculated moxifloxacin effect on myocardial repolarization from E-R analysis was in accordance with that from statistical analyses [34], concentration-effect modelling can be used to clarify ambiguous results and confirm assay sensitivity [27, 29].

Since TQT studies are resource intensive, and E-R analysis evolved to be an important tool in cardiac safety assessment, the question whether early QT assessment using exposure response analysis can replace the TQT study was raised [36]. CRR modelling based upon data from phase 1 study is suggested to be sufficient to identify drug candidates with threshold pharmacology effect [37]. Assuming absence of hysteresis and a linearity of the concentration-response relationship, Darpo et al. applied a linear mixed-effects model, and Emax-model for dofetilide, to examine ∆QTc in time course in relation to concentrations of five “QT-positive” (ondansetron, quinidine, hydrodolasetron, moxifloxacin, dofetilide) and one “QT-negative” drug (levocetirizine) in IQ-CSRC prospective study. Study results met the ICH E-14 criteria putting high confidence on the usage of E-R analysis on ECG and PK data from first-in-human trials and predisposing it to replace the TQT studies [38]. Although TQT studies are still a gold standard in terms of cardiac safety evaluation, the revision of ICH E14 is a hot topic today [37].

“Top-down” modelling is a useful tool also in cardiac toxicity in case of drug poisonings. The toxicokinetic profiles of citalopram [39, 40] and escitalopram [41] were linked to its myocardial repolarization effect with the use of PK/PD modelling. Mégarbane et al. [28] discussed utility and limitations of such models in human acute overdoses. According to the authors, Emax model seems to describe properly PK/PD relationships in cardiotoxic cases. Because of sparse data derived from studies of different scenarios, a population approach is an adequate methodology to meet that issue.

Population pharmacokinetics (PopPK) modelling is not only the valuable tool in toxicology but also throughout the whole drug development process. PopPK identifies and examines the sources of variability in individuals’ drug concentrations. When applied to analyse the phase I of clinical trials data, it should provide good estimates of structural model parameters and establish inter- and intra-individual variability capturing one of the major sources for variable patient response to applied therapy [42]. The PopPK models value raise when there is a link to pharmacodynamic effects described in the form of the PD models. With respect to cardiac safety, the expected range of ∆QTc values may be examined in function of various dosing regimens, and better understanding of benefit/risk ratio in specific populations may be provided. The statistical concept used in population approach include non-linear hierarchical models enabling to investigate variability in patient’s response with respect to covariates such as demographic characteristic as well as taking into account delays in PD response and time-dependent factors in light of which the QTc changes should be interpreted. Furthermore, Bayesian methodologies implemented in PopPK/PD methodology allow the use of prior information in parameter estimation. France and Pasqua provided current and solid review on the PopPK/PD modelling and simulation paradigm in the assessment of QTc interval prolongation suggesting the approach to be a tool for data integration and decision making rather than the methodology of data analysis only [43•].

## “Bottom-Up” Strategy

In contrast to top-down methods, the systems biology “bottom-up” approach requires in-depth mechanistic knowledge of the system which allows integrating molecular level information at a cellular, tissue, or whole-body level, most commonly in a form of mathematical models. Quantitative and systems pharmacology has been defined by the National Institutes of Health as an approach to translational medicine that combines computational and experimental methods to elucidate, validate, and apply new pharmacological concepts to the development and use of small molecule and biologic drugs [1]. Such approach allows for integration and translation of the drug-specific in vitro data to the in vivo human situation. This covers information gathered at the early stages of drug development including safety assessment. In the case of cardiac safety assessments, pure bottom-up modelling and simulation involve reconstruction of processes that define exposure i.e. plasma (or heart tissue) concentration-time profile and its electrophysiological consequences, ideally along with hemodynamic effects and contractility changes. The latter requires models of various complexities from single cell level up to the sophisticated 3-dimensional (3D) multiphase models. Data from a variety of in vitro systems which are surrogates of the in vivo ADME processes allows drug exposure prediction while in vitro information about drug-ion channel interactions enable translation of the exposure to body surface potentials and calculation of electrophysiological endpoints of interest. Separation of drug, system, and trial design data, inherent in bottom-up approach [44, 45], provides a possibility to make a prediction of exposure-response correlation with respect of inter- and intra-individual variability, so it is a useful tool for assessment of drug effect at the population level. Multiple successful examples of physiologically based pharmacokinetic (PBPK) modelling application in drug discovery and development can be found in the literature [46–48]. Much less has been published on physiologically based pharmacodynamic (PBPD) modelling of drug effects specifically for electrophysiological effects in the human heart as it is reported further in the text. The combination of these two approaches is even more rarely reported yet opens a new perspective in the drugs’ safety assessment.

Zemzemi and colleagues presented a study where drug action was projected from ion channel level to the body surface potentials [49]. Three-dimensional anatomical model of human body, including the detailed description of the heart and surrounding tissues, has been developed and joined with a bidomain (extra- and intracellular) electrical model of ventricles, in which ten Tusscher and Panfilov ventricular action potential model was used to represent membrane kinetics [50]. Relevant ionic conductance was modified according to single pore block model to mimic drug-induced potassium and sodium channel block. PK component was not assessed, simply two concentrations for each drug were tested: (i) equal to IC50 and (ii) twice the IC50 value or 10 times of the IC50 value for fast sodium and potassium blockers, respectively. The applied combination of modelling and simulation tools proved to be successful in recovering action potential, ventricle activation, and repolarization alterations; thus, according to the authors, could be used for drug safety assessment based on the prediction of drug effects on QT interval and other ECG-based markers. However, the predictive performance of the tool was tested solely for single channel block at a time with no variability introduced; thus, it requires further investigations to prove its utility in drug safety testing.

Obiol-Pardo proposed a combination of multiple modelling and simulation techniques forming system for the drugs proarrhythmic properties prediction [51]. The combination of the 3D-QSAR model and cellular (1D) and tissue (2D) levels ventricular electrophysiological models allowed for extrapolation from the level of chemical structure up to the clinical endpoint (QT prolongation). Multiple concentrations for four included compounds were tested. Results were presented as maps showing a correlation between QT prolongation and in silico predicted potassium ionic channels inhibition. Okada and colleagues proposed a similar approach based on the complex 3D finite element method (FEM), based human heart model and assessed the effect of 12 benchmark drugs on simulated ECG signal [52]. In vitro characterization of these substances included assessment of inhibitory effects on the six main cardiac ion currents/channels. Multiple active concentrations were tested and they represented values equal to 0 (control), 1, 3, 5, 10, 30, 50, 100, 300, 500, and 1000 times of free effective therapeutic plasma concentration. The simulated, drug-specific ECG signals were analyzed to obtain the concentration threshold values for which arrhythmia occurred. They were further compared against the TdP risk categories proposed by Redfern [53]. Authors claim that they were able to successfully identify the drug category in most cases and avoid false positives. The abovementioned approaches did not offer parallel pharmacokinetics prediction. The inter-individual variability was not analyzed either. There are only a few examples where both components of drug interaction, namely, PK and PD, were modelled and simulated in parallel in order to provide an evaluation of drug cardiac effect. The first report, where a concept of combined PBPK/PD modelling and simulation to predict bedside cardiac effects of drugs was coined, describes PK and PD mechanistic models operating exclusively on in vitro data used to predict QTc prolongation [54]. Quinidine was chosen as a model drug due to its known cardiac effect and relatively rich in vitro and in vivo data available in the literature. The population-based Simcyp Simulator was used to simulate the individual free plasma concentrations of quinidine and its main metabolite 3-hydroxyquinidine. They were further utilized together with the literature derived IC50 values for multiple ionic channels as the inputs for the ten Tusscher et al. model describing human cardiac ventricular cell electrophysiology. Single cells were virtually connected to the one-dimensional heterogeneous string to mimic the human left heart wall, and crucial physiological parameters were randomly assigned for virtual individuals to mimic real life variability. The simulated endpoints (concentration vs time for the PK and QTc or ΔQTc changes vs time for the PD part) were further compared against their clinical counterparts, and the obtained results showed a high level of consistency. As evidenced by the study by Mishra et al., where electrophysiological consequences of concomitant administration of drug with its metabolic inhibitor were simulated, combining QSAR methods, PBPK and PBPD modelling, and systems biology can be used to do the cardiac safety assessment [55]. By taking into account not only average maximal effective concentrations of a drug but also those resulting from an overdose, drug–drug interaction or active metabolites such analysis becomes more robust.

Systems pharmacology or “middle-out” approach sits at the interface between the other two discussed categories. These combine aspects of both PK/PD and systems biology, and incorporate physiological processes and mechanism of action at targets [56] and, therefore, allow for effective use of all available data. Examples of its practical utilization include recent publications, where information about drug plasma concentration gathered from clinical trials was combined with biophysically detailed models of cardiac cells electrophysiology. Ultimate aim was to simulate QT prolongation for various antipsychotic drugs [57, 58]. Mirams and colleagues used a similar approach with the aim to predict TQT trials results yet their simulation strategy involved single cell models [59]. Therefore, the study endpoint was defined as the action potential duration modification.

## Conclusion

Application of modelling and simulation in the proarrhythmic potency assessment area has been a standard procedure for a long time. It includes research and development as well as regulatory decision levels. Review of the clinical study with ECG-derived endpoints by default includes an assessment of the concentration-effect relationship analysis. For drugs that affect QT, the approval decisions are based to a large extent on dose- and concentration-QT relationships [60]. The model-based testing approach is likely to change the paradigm in how to assess cardiac safety. Thorough analysis and interpretation of the multidimensional in vitro data to characterize inhibition of various ionic channels require appropriate tools and biophysically detailed models of human cardiac electrophysiology allow quantitative extrapolation of the cardiac effect, which can assist the design of clinical safety assessment studies in combination with PBPK and PBPD modelling.
